# Predatory Mechanisms of Myxobacteria and Their Potential in the Development of Biocontrol Agents

**DOI:** 10.4014/jmb.2608.08010

**Published:** 2026-09-21

**Authors:** Bongsoo Lee

**Affiliations:** Department of Microbial Biotechnology, Mokwon University, 88 Doanbuk-ro, Seo-gu, Daejeon 35349, Republic of Korea

**Keywords:** Myxobacteria, Predation, Secondary metabolites, Biocontrol

## Abstract

Climate change, environmental degradation, and the depletion of finite resources are intensifying the search for sustainable, bio-based solutions across agriculture, environmental management, and chemical production. Against this backdrop, myxobacteria have emerged as a valuable but still underused microbial resource, distinguished by their ecological versatility, multicellular behavior, predatory lifestyle, and biosynthetic capacity. This review integrates the mechanistic basis of myxobacterial predation with its translational potential in biocontrol, linking microbial ecology to applied biotechnology. The available evidence indicates that predation is not a single process but a coordinated program built from swarm behavior, gliding motility, contact-dependent killing, secreted hydrolytic enzymes, outer membrane vesicle (OMV)-mediated interference, nutrient competition, and specialized metabolites. Through these layers, myxobacteria antagonize phylogenetically diverse targets, including bacteria, fungi, oomycetes, and ciliates, with more limited evidence extending to cyanobacteria and nematodes. Unlike recent reviews that address either the cellular and genetic mechanisms of predation or antagonism against plant pathogens in isolation, this review connects mechanism with application and compares the maturity of the available evidence across target classes. Within this framework, selected bacterial and fungal plant-disease systems have field-level support; oomycetes are mechanistically well supported but have limited field validation; ciliates provide direct mechanistic predation evidence without biocontrol validation; cyanobacteria remain emerging with laboratory-based evidence; and nematodes remain preliminary, with evidence restricted to *in vitro* studies using *Caenorhabditis elegans*. Myxobacteria therefore merit continued investigation not only as model organisms for microbial ecology and natural product discovery but also as multifunctional platforms for sustainable biocontrol and broader biotechnological applications.

## Introduction

Continued dependence on fossil fuels has intensified pollution and anthropogenic warming [1, 2], while climate change increasingly threatens biodiversity, public health, and food security [2, 3]. In parallel, depletion of finite resources has strengthened demand for sustainable, bio-based alternatives [4, 5], including microbial platforms for bioremediation [6], crop protection [7], and value-added chemical production [4, 5]. One visible consequence of warming and eutrophication is the increasing frequency and severity of harmful algal blooms (HABs), particularly cyanobacterial blooms in inland waters [8, 9]. Such blooms can degrade water quality and disrupt aquatic ecosystems [9, 10], while cyanotoxin production poses risks to wildlife and human health [10, 11]. These concerns have stimulated interest in biological mitigation strategies, including algicidal bacteria [12, 13].

Climate change also threatens agricultural sustainability by altering crop production and the distribution and impact of pests and pathogens [14]. Plant-parasitic nematodes are major soil-borne pests that cause substantial crop losses [15, 16] and pose risks to food security [17]. Climate change may further alter their distribution and activity [17] and increase crop damage in some nematode–crop systems [18]. Given the substantial agricultural losses associated with these pests [15, 19], biological control strategies that reduce reliance on chemical nematicides are increasingly important [19, 20]. In parallel, advances in microbial engineering have expanded the use of microbial cell factories for bio-based production [4, 5], including engineered biomaterials [21]. Among these microbial resources, myxobacteria are notable for their complex multicellular and predatory behaviors [22] and exceptional biosynthetic capacity [23]. The best-characterized species, *Myxococcus xanthus*, has become a leading model for studying social interactions, multicellular development, and bacterial predation [24]. Myxobacteria are also prolific producers of structurally diverse secondary metabolites with antibacterial, antifungal, antiviral, cytotoxic, and other bioactivities [23, 25], and are recognized alongside actinomycetes as important bacterial sources of novel natural products and anti-infective leads [26]. In *M. xanthus*, predation is a multifactorial process involving coordinated motility, extracellular enzymes and metabolites, and contact-dependent killing [27, 28]. Recent studies in *M. xanthus* have identified Tad-like and atypical type III-like systems, including adaptable Tad-pilus tips, that mediate contact-dependent prey killing [29-31]. These findings broaden the earlier extracellular-centered view of predation and support a model in which extracellular and contact-dependent mechanisms act in concert [28].

Myxobacterial predatory and antagonistic interactions extend beyond bacteria to selected eukaryotic microorganisms. Specialized carbohydrate-active enzymes mediate predation on oomycetes [32], whereas tubulysins are required for ciliate killing in defined myxobacterial systems [33]. In plant pathology, myxobacterial metabolites, extracellular enzymes, and predatory or antagonistic activities have been reported against fungal, oomycete, and bacterial phytopathogens [34, 35]. These findings, together with the broader ecological potential of predatory bacteria, have stimulated interest in myxobacteria as biocontrol resources [36]. Recent work has demonstrated the biocontrol activity of *Myxococcus fulvus* WCH05 against fire blight [35], while expanding genomic, natural-product, and biodiversity resources continue to support the discovery and characterization of myxobacterial diversity [37-39]. Recent reviews have addressed distinct aspects of this field, including the cellular and ecological mechanisms of predation in *M. xanthus* [27], the genetic basis of predatory killing [28], and the active substances involved in antagonism against plant pathogens [34]. However, an integrated assessment linking predatory mechanisms with biocontrol applications across different target groups remains lacking. This review therefore has two complementary aims: to synthesize the major components of myxobacterial predation, including cooperative swarming, contact-dependent killing, secreted hydrolytic enzymes, outer membrane vesicle-mediated interference, specialized metabolites, and nutrient competition; and to comparatively assess the maturity of the available evidence across target groups. Among the target groups considered, field-oriented evidence is currently strongest in selected bacterial and fungal plant-disease systems. Oomycetes have strong mechanistic and rhizosphere-level support but require broader field validation; ciliates provide direct mechanistic predation evidence without applied biocontrol validation; bloom-forming cyanobacteria remain an emerging environmental target supported primarily by laboratory studies; and nematode-related evidence remains preliminary and limited to *in vitro* studies using the model nematode *Caenorhabditis elegans*. This evidence-based framework is intended to clarify both the biological potential of myxobacteria and the strength of the evidence supporting their development as biocontrol agents.

### Myxobacteria: Taxonomy, Multicellular Life Cycle, and Biosynthetic Potential

Myxobacteria are rod-shaped, Gram-negative bacteria defined by an unusual combination of surface-associated multicellular behavior, coordinated social motility, predatory growth, and starvation-induced fruiting body development [22]. Molecular phylogeny established them as a coherent lineage, and genome-based reclassification has since placed them within the phylum Myxococcota, reflecting their deep evolutionary distinctiveness and expanding diversity [40, 41]. Their biology and their chemistry are connected: the large genomes that support complex social behavior also encode a rich biosynthetic gene repertoire [40]. Recent genomic and biodiversity-oriented resources indicate that this phylogenetic and biosynthetic diversity is still growing, and that currently characterized taxa likely represent only a fraction of the group's accessible ecological and biotechnological potential [37, 39].

The myxobacterial life cycle is among the most sophisticated known in bacteria, combining vegetative growth under nutrient-replete conditions with starvation-induced multicellular development [22]. In *M. xanthus*, the best-characterized model, starvation triggers coordinated aggregation and differentiation into fruiting bodies containing environmentally resistant myxospores [24]. Development is therefore not merely a morphological process but a regulated survival strategy that couples starvation-induced differentiation with subsequent recovery when favorable conditions return [24, 42]. Importantly, starvation-induced morphogenesis in *M. xanthus* produces multiple cell fates rather than a single outcome, including myxospores, peripheral rods, and cells that undergo developmental lysis [43, 44]. Together with its social organization and predatory ecology, these developmental transitions have made *M. xanthus* an important model for multicellularity, differentiation, collective decision-making, and prokaryotic competition [24]. Beyond this multicellular behavior, myxobacteria are renowned for their biosynthetic ability. Their comparatively large genomes encode extensive regulatory networks, hydrolytic enzymes, secretion systems, and numerous biosynthetic gene clusters that together support predation, environmental adaptability, and specialized metabolism [40]. Myxobacterial chemical diversity continues to expand through genome mining, metabolomics, and database-assisted discovery [25, 37, 38, 45]. The metabolites span antibacterial, antifungal, antiparasitic, antiviral, and cytotoxic activities, and many display novel scaffolds and modes of action [23]. For these reasons, myxobacteria are widely regarded, alongside actinomycetes, as one of the most important microbial sources of new antibiotics and other high-value natural products [26].

***M. xanthus* as a model organism for multicellular development and cooperative predation.** *M. xanthus* has become the principal model for studying multicellular development, social motility, and cooperative predation [24]. It is commonly found in soils and other organic-matter-rich terrestrial habitats, where it obtains nutrients by degrading extracellular organic material or by preying on neighboring microorganisms [27]. During vegetative growth, cells often move in coordinated groups and feed collectively, a behavior historically described as wolf-pack predation, in which high local cell density can promote the accumulation of extracellular lytic activities and the release of nutrients accessible to the swarm [46]. Such group behavior can enhance extracellular digestion and nutrient acquisition, although individual *M. xanthus* cells are also capable of killing prey (Fig. 1) [46]. When nutrients become limiting, *M. xanthus* undergoes a striking developmental transition in which swarming populations aggregate and differentiate into fruiting bodies filled with resistant myxospores [24]. As noted above, this program generates multiple cell fates: some cells form spores, while others persist as peripheral rods or undergo developmental lysis [43, 44]. The fruiting body thus functions as more than a spore container; its characteristic multicellular architecture can influence spore recovery following specific environmental stresses [42]. A key feature of the cycle is that sporulation is tightly coupled to renewed predation. When nutrients or prey return, myxospores germinate into motile vegetative rods that re-establish coordinated swarming and resume cooperative feeding [24]. The life cycle of *M. xanthus* is therefore best understood not as a simple alternation between growth and dormancy but as a cyclical system linking starvation survival to the reactivation of wolf-pack predation, as illustrated schematically in Fig. 1 [46].

**Gliding motility in *M. xanthus*: relevance to development and predation.** Gliding motility is central to both the vegetative and developmental phases of the *M. xanthus* life cycle, because surface translocation is required for swarm expansion, prey encounter, aggregation, and fruiting body morphogenesis [47]. Two genetically separable forms have historically been described: adventurous (A) motility, which drives individual cell movement, and social (S) motility, which underlies coordinated group migration [48]. The terminology remains in use, but the mechanistic picture has sharpened. A-motility is associated with a focal-adhesion-like gliding apparatus built around the Agl-Glt machinery, which assembles dynamically along the cell envelope and generates traction for single-cell movement [49, 50], and recent work has identified molecular regulators that control the assembly of these focal adhesion complexes [51]. S-motility, by contrast, depends mainly on type IV pili and the extracellular matrix — particularly exopolysaccharides — which together promote cell-cell cohesion and coordinated surface movement [52, 53].

For the present review, the key consideration is not the full mechanistic detail of motility but its functional significance. Gliding motility enables *M. xanthus* populations to organize at the prey interface, maintain high local cell density, and optimize the cooperative deployment of lytic enzymes and bioactive metabolites [27, 47]. Motility is therefore inseparable from predation, providing the behavioral framework that allows populations to search for prey, invade prey colonies, and convert released nutrients into swarm expansion. The same coordinated surface behaviors are repurposed during starvation to drive aggregation and development [47], making motility an integrating mechanism linking vegetative growth, cooperative predation, and developmental morphogenesis.

**Secondary metabolites and bioactive potential of myxobacteria.** Myxobacteria biosynthesize structurally diverse secondary metabolites and other bioactive molecules at a level matched by few bacterial groups [23, 25]. For nearly two decades, they have been recognized as one of the richest sources of chemically unique natural products, and they are now regarded as microbial natural product factories of exceptional value to drug discovery [54]. Historical surveys catalogued dozens of scaffold classes and hundreds of structural variants [23]. Genome-guided and metabolomics-based approaches indicate that the accessible chemical space is far from exhausted [25, 37, 38, 45]. The comparison with actinomycetes is justified because both groups have repeatedly yielded structurally unprecedented metabolites with novel activities and mechanisms [26]. Large-scale comparative metabolomics has further shown that chemical diversity tracks taxonomic distance in myxobacteria, which makes less-studied genera particularly promising for the discovery of new metabolite families [55]. A few emblematic scaffolds illustrate this breadth. Epothilones, microtubule-stabilizing macrolides first isolated from *Sorangium cellulosum*, are notable because their mechanism resembles that of paclitaxel while retaining activity against certain drug-resistant tumor cells, which inspired clinically relevant analogs such as ixabepilone (BMS-247550) [56-58]. Tubulysins are another landmark class: these exceptionally potent tubulin-interfering peptides show picomolar-range cytostatic activity and have long served as scaffolds for anticancer development [59, 60]. Disorazoles, highly cytotoxic macrodiolides from *S. cellulosum*, exert potent tubulin-directed antimitotic activity [61], whereas chivosazoles from the same species disrupt the actin cytoskeleton, further illustrating the breadth of cytoskeleton-targeting myxobacterial chemistry [62]. Sorangicin A, also from *S. cellulosum*, is a macrolide antibiotic that inhibits bacterial RNA polymerase and remains a key example of transcription-targeting anti-infective chemistry [63], and myxalamides from *M. xanthus* add respiratory-chain inhibition to this functional range [64].

Several myxobacterial antibiotics act on bacterial transcription. Myxopyronin and corallopyronin inhibit bacterial RNA polymerase by binding to the switch region, a mechanistically distinctive mode of action [65]. Corallopyronins were originally isolated from *Corallococcus coralloides* and characterized as inhibitors of bacterial RNA synthesis [66]. Corallopyronin A has subsequently advanced as a preclinical anti-*Wolbachia* candidate for antifilarial applications [67]. Cystobactamids have emerged as promising leads against Gram-negative pathogens owing to their unusual gyrase/topoisomerase-directed activity [68], and haliangicin, a marine myxobacterial antifungal, continues to draw attention as a distinctive β-methoxyacrylate natural product [69]. More recently described metabolites from less conventional taxa, such as the aetheramides from *Aetherobacter*, underscore how much chemically relevant diversity remains to be explored [70, 71].

The ecological significance of these compounds should, however, be distinguished from their pharmacological value. Not all pharmacologically important myxobacterial metabolites have demonstrated roles in predation. Nevertheless, some specialized metabolites function directly as prey-selective predatory weapons. In *M. xanthus*, disruption of antibiotic TA (myxovirescin A) biosynthesis impaired predation on *Escherichia coli* but not on *Micrococcus luteus*, while the same study linked corallopyronin A production by *C. coralloides* to efficient *E. coli* predation [72]. Accordingly, myxobacterial specialized metabolism should be understood both as a source of compounds with translational value and as a functional component of predatory ecology. Representative metabolites, together with their producing taxa, major bioactivities, and ecological or translational relevance, are summarized in Table 1.

### Predatory Mechanisms of Myxobacteria

Predation is a defining ecological trait of myxobacteria and contributes to their success in densely populated microbial habitats [27]. Unlike obligate bacterial predators that depend entirely on prey for growth, myxobacteria are facultative predators that can grow saprophytically on nonliving organic substrates while actively killing and consuming living prey under favorable conditions [73]. Myxobacterial predation was historically viewed primarily as an extracellular process mediated by cooperatively deployed lytic enzymes and toxic metabolites [72, 74]. More recent studies have expanded this view by demonstrating the involvement of direct cell contact, specialized secretion systems, outer-membrane-associated factors, nutrient competition, and prey-specific molecular adaptations [28, 75]. Predatory success also depends on broader physiological responses, including metabolic reorganization and stress adaptation [76]. These observations frame myxobacterial predation as a multilayered process rather than a single mechanism. The major components are examined in the following subsections.

**Cooperative predation and prey range.** A defining feature of myxobacterial predation is its frequently cooperative nature. Swarming cells accumulate at prey interfaces and collectively deploy factors whose effectiveness increases at high local cell density [27]. This behavior provides an ecological advantage in structured habitats such as soil and rhizosphere-associated microbial communities [46]. By concentrating extracellular enzymes, antibiotics, and membrane-associated effectors at the prey surface, myxobacterial swarms enhance prey killing and nutrient acquisition [27, 72]. Cooperation is not obligatory in every context, however, because individual cells or small groups can also contribute substantially to contact-dependent prey killing [28, 75].

Myxobacteria exhibit an exceptionally broad prey spectrum and can attack both Gram-positive and Gram-negative bacteria. Comparative analyses of *Myxococcus* predation across diverse bacterial species further indicate that prey identity and Gram status strongly influence predatory performance and predator growth [77]. Myxobacterial interactions also extend to selected eukaryotic microorganisms, including fungi, oomycetes, and ciliates [28, 32, 33]. Within these systems, the available evidence ranges from direct mechanistic predation to broader antagonistic activity. This breadth is relevant to biocontrol potential because traits that suppress microbial competitors in natural communities may also act against agriculturally or environmentally harmful targets [34, 36]. Prey recognition and choice are increasingly viewed as dynamic and context-dependent rather than constitutive. Genetic and ecological evidence indicates that predatory outcomes vary with prey identity and environmental conditions [28], whereas comparative genomic analyses of *Corallococcus* spp. reveal substantial plasticity in predation-associated gene repertoires [78]. These findings suggest that myxobacterial predatory capacity is shaped by both immediate ecological context and lineage-specific genomic potential. Prey organisms are not passive during these interactions; exposure to myxobacteria can alter secondary metabolism and developmental morphology in susceptible prey [79]. Predation should therefore be understood as a dynamic interaction rather than a one-sided lytic event. It also occurs within a broader framework of social conflict, in which kin discrimination influences antagonistic cell–cell interactions among myxobacteria [80].

**Contact-dependent killing systems.** One of the most important recent advances in myxobacterial predation research is the recognition that prey killing is not mediated solely by diffusible lytic factors. Direct cell contact can contribute substantially to prey attack in at least some myxobacterial systems; in *M. xanthus*, a Tad-like apparatus known as the Kil system is required for efficient contact-dependent prey killing [29]. Subsequent studies further resolved the molecular basis of this process. *M. xanthus* was shown to combine the Tad-like Kil system with an atypical type III-like protein secretion system during bacterial prey killing [30]. More recent work demonstrated that the Kil machinery polymerizes a KilP-containing Tad pilus and that adaptable minor-pilin tip complexes mediate prey-dependent contact and killing [31]. These findings establish a functional association between prey engagement, Kil-pilus assembly, and the atypical type III-like system, while indicating distinct contributions to prey killing and lysis [30, 31].

Contact-dependent killing complements rather than replaces extracellular attack in the characterized systems described above. Together with broader evidence summarized across myxobacteria, these findings support a hybrid model in which diffusible enzymes and metabolites act at the community level, whereas contact-associated machinery enables localized prey engagement and killing [28]. Surface motility supports this process by bringing predators into contact with prey and organizing the spatial interactions required for localized effector activity and collective feeding [27, 47]. Movement, prey contact, and killing should therefore be viewed as functionally integrated components of the broader predatory program [75]. Contact-mediated interactions are also relevant to social recognition and kin discrimination. Predation and kin discrimination represent distinct ecological reasons for killing, although both operate within the wider framework of multicellular interaction and competition in myxobacteria [80].

**Secreted enzymes, outer membrane vesicles, and specialized metabolites.** Extracellular digestion remains a core component of myxobacterial predation alongside contact-dependent killing [74]. These enzymatic systems can be highly specialized when myxobacteria attack fungal and oomycete prey. An outer-membrane β-1,6-glucanase involved in fungal predation enables myxobacteria to target a structural component of the fungal cell wall [81]. Predation on oomycetes similarly depends on a specialized carbohydrate-active enzyme (CAZyme) repertoire shaped by adaptive evolution, providing evidence that prey-specific enzymes can contribute directly to predation on eukaryotic plant pathogens [32]. Beyond these characterized systems, chitinases, proteases, and peptidases have also been implicated in myxobacterial antagonism toward plant pathogens, although their specific contributions to predation remain incompletely resolved [34]. Outer membrane vesicles (OMVs) provide another mechanistic layer in ecological interference. A particularly clear mechanistic study showed that myxobacteria restrain *Phytophthora sojae* by delivering thiaminase I via OMVs. The enzyme depletes extracellular thiamine in the soybean rhizosphere, thereby limiting the pathogen’s access to this essential public metabolite [82]. Prey suppression can therefore occur through vesicle-mediated nutrient interference without requiring direct cell lysis. Evidence that OMVs directly mediate predator–fungus interactions further supports their role in myxobacterial predation on eukaryotic prey [83].

Specialized metabolites constitute another major component of the predatory arsenal. Myxobacterial natural products display antibacterial, antifungal, antiparasitic, and cytotoxic activities [23], and some have experimentally demonstrated ecological functions. Antibiotic production contributes directly to bacterial prey killing [72], whereas tubulysins are required for efficient predation on ciliates, linking a well-characterized bioactive metabolite to the suppression of eukaryotic prey [33]. The relative contributions of these mechanisms vary with prey identity, environmental conditions, and the balance between extracellular attack and close-range engagement [28, 73]. Nutrient competition can also influence predatory outcomes; siderophore-mediated competition for iron substantially alters prey susceptibility and predation dynamics [84]. Myxobacterial predation is therefore best understood as an integrated and context-responsive process involving coordinated swarming, contact-dependent killing, hydrolytic enzymes, OMV-mediated interference, specialized metabolites, nutrient competition, and physiological adaptations that sustain predatory performance [27, 72, 76, 82, 84]. The major mechanistic modules of myxobacterial predation and their relationship to ecological and biocontrol outcomes are summarized in Fig. 2.

### From Predation to Biocontrol Applications

As illustrated in Fig. 2, the translational potential of myxobacteria derives from the same traits that support their predatory lifestyle, including cooperative surface colonization, extracellular hydrolytic activity, OMV-mediated interference, and structurally diverse bioactive metabolites [27, 34]. These attributes have positioned myxobacteria as promising candidates for sustainable biocontrol in agricultural and environmental systems [34, 36]. Activity under simplified laboratory conditions, however, does not by itself establish practical biocontrol performance. Effective application in complex environments also depends on colonization, persistence, ecological compatibility, formulation, and delivery [85, 86]. The following subsections are organized according to the strength and maturity of the available evidence rather than as a simple catalog of target organisms. Relevant studies for each target group were reviewed and organized according to the type and maturity of the evidence they provided. Evidence maturity was then assessed qualitatively using four criteria: mechanistic resolution, experimental context, breadth of support across strains, targets, or pathosystems, and validation under rhizosphere, pot, greenhouse, or field conditions. Within this framework, “field-supported” denotes evidence extending to crop-level field trials in one or more defined pathosystems; “mechanistically well supported” denotes defined molecular or ecological mechanisms demonstrated in laboratory, rhizosphere, or pot systems but with limited field validation; “emerging” denotes predominantly laboratory-based antagonism without field-scale demonstration; and “preliminary” denotes limited evidence derived from extract-based assays, model organisms, or isolated reports. These categories refer to the specific pathosystems or experimental models represented by the available studies and should not be interpreted as applying uniformly to entire target groups. Ciliates are assigned a separate descriptive category because direct mechanistic prey killing has been demonstrated, whereas applied biocontrol efficacy has not been validated. Accordingly, selected bacterial and fungal plant-disease systems currently provide the strongest field-oriented evidence; oomycetes are mechanistically well supported but remain limited in field validation; ciliates provide direct mechanistic predation evidence without applied biocontrol validation; cyanobacteria remain emerging targets supported primarily by laboratory evidence; and nematodes remain preliminary, with evidence restricted to *in vitro* studies using the model nematode *C. elegans*. This target-group evidence hierarchy is summarized in Table 2.

**Plant-pathogenic bacteria and fungi.** Plant-pathogenic bacteria and fungi are major targets for microbial biocontrol because they cause substantial crop losses, quality deterioration, and economic damage across agricultural systems [85–88]. Long-term reliance on chemical bactericides and fungicides is increasingly constrained by environmental concerns, regulatory pressure, and resistant pathogen populations [86-88], which has driven the search for sustainable microbial antagonists as alternatives or complements to conventional disease management [85, 86]. Myxobacteria are attractive in this context because they combine predatory interactions, extracellular enzymatic activity, and metabolite-mediated antagonism against a broad range of phytopathogens.

A recent review concluded that myxobacteria can inhibit plant-pathogenic fungi, oomycetes, and bacteria through extracellular enzymes, small-molecule metabolites, and predatory behavior, identifying plant disease control as one of the most promising practical applications of the group [34]. Screening studies have reinforced this potential, showing that multiple strains inhibit phytopathogenic fungi and oomycetes *in vitro*, with inhibition patterns that differ among targets and suggest distinct contributions of contact-associated activity and diffusible factors [89]. Earlier application-oriented studies also provided evidence in selected fungal plant-disease systems. Culture filtrate of *Myxococcus* sp. KYC 1126 suppressed *Colletotrichum acutatum*-induced anthracnose in hot pepper in greenhouse and field assays, although field efficacy varied between years and was not sustained over prolonged periods [90]. Culture filtrate of *S. cellulosum* KYC 3270 also suppressed *Botrytis cinerea* gray mold on stored fruits [91]. A subsequent three-year field trial demonstrated suppression of hot-pepper anthracnose by *S. cellulosum* KYC 3262 culture filtrate, providing longer-term field evidence in this selected fungal pathosystem [92].

Field-level evidence of fungal wilt suppression is available in cucumber. A predatory myxobacterium controlled cucumber *Fusarium* wilt in greenhouse and two-year field experiments by migrating toward plant roots, reducing pathogen abundance, and reshaping the rhizosphere microbial community, indicating that suppression involves microbiome-level restructuring in addition to direct antagonism [93]. Comparable evidence exists for bacterial wilt. *M. xanthus* R31 showed strong antagonism against *Ralstonia solanacearum*, and pot experiments demonstrated substantial suppression of tomato bacterial wilt. Secreted protein fractions, rather than ethyl acetate extracts, were primarily responsible for pathogen inhibition, indicating an important role for extracellular proteinaceous factors [94].

Translational potential also extends to cotton Verticillium wilt. *Cystobacter fuscus* HM-E suppressed disease in greenhouse pot experiments, and a formulated solid agent showed greater efficacy than the fermentation broth, highlighting the importance of application format [95]. A complementary study demonstrated that *Myxococcus* sp. MP20 delivered myxothiazole to *Verticillium dahliae* through OMVs, thereby inhibiting fungal growth and suppressing cotton Verticillium wilt [83]. Disease suppression has more recently been extended to wheat Fusarium crown rot. A 2026 study screened 81 myxobacterial strains, identified highly active antagonists against the predominant crown-rot pathogens, and validated their efficacy in greenhouse and field experiments [96]. By linking broad strain selection with greenhouse and field testing, this study provides one of the strongest current demonstrations that myxobacterial antagonists can be translated into crop-level disease suppression [96]. The progression from strain validation to field-oriented production is illustrated by *M. fulvus* WCH05. Following the initial demonstration of WCH05-mediated fire blight suppression in detached inflorescence and greenhouse assays [35], optimization of its fermentation conditions increased biomass production and improved biocontrol efficacy under greenhouse and field conditions [97]. Collectively, these studies indicate that selected bacterial and fungal plant-disease systems currently provide the strongest field-oriented evidence for myxobacterial biocontrol [90, 92, 93, 96, 97].

**Oomycete pathogens.** Oomycetes deserve separate attention because they include destructive causal agents of root rot, damping-off, and blight, particularly species of *Phytophthora* and *Pythium*, and are phylogenetically and biologically distinct from fungi despite their morphological resemblance [98]. Myxobacteria can interact with these pathogens through both prey-adapted enzymatic systems and ecological interference mechanisms. A 2023 study demonstrated that myxobacterial predation on oomycetes depends on a specialized carbohydrate-active enzyme (CAZyme) repertoire shaped by adaptive evolution, providing direct mechanistic evidence that predation can be prey-type specific rather than purely generalist [32]. A second key study showed that myxobacteria restrain *P. sojae* in the soybean rhizosphere by delivering thiaminase I through outer membrane vesicles. The resulting depletion of extracellular thiamine limits pathogen growth and invasion, demonstrating that suppression can occur through nutrient interference rather than direct lysis [82]. In addition, screening studies have shown that selected myxobacterial strains inhibit oomycetes such as *Pythium ultimum* *in vitro*, further supporting oomycetes as realistic targets of myxobacterial antagonism [89]. Application-oriented evidence is also available from a greenhouse study of hot-pepper Phytophthora blight. Culture filtrate of *Myxococcus* sp. KYC 1126 suppressed disease caused by *Phytophthora capsici*, with efficacy differing between phyllosphere and rhizosphere applications [99]. However, phytotoxicity was observed under prolonged exposure to the culture filtrate, indicating that application conditions require careful optimization.

Together, these studies establish oomycetes as one of the most mechanistically well-supported target groups for myxobacterial biocontrol, with additional application-oriented evidence from greenhouse studies. However, field validation remains limited, and broader evaluation is needed to determine whether this evidence can be extended to a level comparable to that demonstrated in selected bacterial and fungal plant-disease systems [32, 82, 89, 99].

**Ciliates: mechanistic predation and ecological relevance.** Protozoa and ciliates are not conventional crop pathogens but are important components of soil and aquatic microbial food webs that, through bacterial grazing and rhizosphere interactions, influence nutrient mineralization, bacterial turnover, and the assembly of plant-associated microbial communities [100, 101]. They are therefore relevant not as direct disease targets but as ecological regulators whose manipulation may have implications for microbiome engineering, soil health, and water management. Among eukaryotic prey, ciliates are currently supported by the clearest direct mechanistic evidence. Yu et al. demonstrated that tubulysins are required for efficient predation of *Tetrahymena thermophila* by *Archangium gephyra* KYC5002, directly linking a specialized metabolite to eukaryotic prey killing [33]. This study provides direct evidence that a myxobacterium can use a specialized metabolite for predation on a microbial eukaryote. At present, ciliate predation provides direct mechanistic evidence for eukaryotic prey killing without demonstrated biocontrol application [33].

**Cyanobacteria and the control of bloom-forming phototrophs.** Bloom-forming cyanobacteria are important potential targets for biological control because they disrupt aquatic ecosystem function, reduce water quality, and may produce cyanotoxins that threaten wildlife, livestock, and human health [8–10, 102, 103]. As warming, water-column stratification, and nutrient enrichment increasingly favor bloom formation and persistence, cyanobacterial proliferation has become a growing concern for water security [8, 9, 11]. These pressures have intensified interest in ecologically informed mitigation strategies, including the use of algicidal microorganisms [12, 13]. The application of myxobacteria against cyanobacteria is scientifically promising but remains less mature than the evidence available in selected plant-disease systems. Bauer and Forchhammer reassessed bacterial predation on cyanobacteria within a phylogenetic framework and discussed myxobacterial interactions with cyanobacterial prey, while cautioning that some earlier reports are affected by taxonomic ambiguity and should therefore be interpreted conservatively [104].

Direct experimental evidence is available from classical laboratory studies. Burnham et al. demonstrated myxococcal predation of *Phormidium luridum* in aqueous environments [105], and Daft et al. subsequently reported lysis by *M. fulvus* under continuous-flow conditions [106]. These studies provide direct laboratory evidence of predation and lysis of cyanobacterial prey by selected myxobacteria [105, 106]. Reliable suppression of natural, field-scale blooms has yet to be demonstrated, placing cyanobacteria among the emerging targets in this application framework [104].

**Nematodes: early evidence, potential, and current limitations.** Plant-parasitic nematodes are important targets for biological control because they cause substantial yield and quality losses across diverse crops and include economically important root-knot, lesion, and cyst nematodes [15, 16, 107, 108]. Nematode-related damage also extends beyond agriculture; the pine wood nematode, for example, is a major threat to forest ecosystems and timber production [109]. These impacts have increased the need for sustainable management strategies, particularly as the use of conventional chemical nematicides becomes increasingly restricted [20, 107].

Evidence for the antinematodal potential of myxobacteria remains preliminary. A 2022 study evaluated metabolite extracts from ten myxobacterial isolates against the model nematode *C. elegans* and reported substantial mortality under *in vitro* conditions [110]. More recently, Hyun et al. showed that *Myxococcus stipitatus* DSM14675 strongly suppressed *C. elegans* under DKxanthene-producing co-culture conditions, whereas nematodes proliferated under conditions in which DKxanthenes were not produced or when DKxanthene-deficient mutants were used. Partially purified DKxanthene extracts also inhibited *C. elegans* growth, providing more specific evidence that these metabolites contribute to antinematodal activity [111].

Taken together, these findings support preliminary antinematodal activity in an *in vitro* model-nematode system. However, the available evidence does not yet demonstrate direct predation on nematodes or effective control of agriculturally important plant-parasitic species. Practical control of plant-parasitic nematodes therefore remains unvalidated [107, 110, 111].

**Opportunities and bottlenecks for field application.** The available evidence indicates that myxobacteria have clear translational potential, although field deployment remains a major bottleneck [36, 112]. Research on plant diseases has progressed beyond laboratory antagonism to rhizosphere-mediated suppression and pot or greenhouse studies, with field validation reported in selected crop pathosystems [93–97]. These advances mark an important transition from conceptual potential to applied evaluation. However, the biological traits that support myxobacterial predation may also complicate product development. Their collective behavior, dependence on surface-associated growth, and context-dependent predatory performance can contribute to variable efficacy across soils and plant-associated environments [27, 85]. Importantly, translational strategies should distinguish live-cell biocontrol from cell-free approaches. Live-cell applications depend on the production, preservation, delivery, establishment, and persistence of viable predatory cells, whereas cell-free products—including extracellular proteins, OMV-delivered enzymes or metabolites, fermentation-derived products, and purified bioactive compounds—require separate consideration of formulation stability, activity retention, delivery, biosafety, and regulatory requirements [82, 83, 94, 95]. These application routes differ in their requirements for formulation, stability, ecological safety, and regulatory evaluation [85, 86, 112].

Field-oriented microbial biocontrol studies emphasize the importance of environmental persistence, scale-up, and compatibility with the target agroecosystem [85, 86, 112], while myxobacterial case studies show that fermentation optimization can improve biomass production and biocontrol performance [97]. Reliable applications will therefore require application-specific integration of cultivation or production, formulation, ecological safety assessment, and multi-location field validation [112]. The major target groups discussed in this section, together with their representative mechanisms, experimental support, and current evidence levels, are summarized in Table 2.

## Conclusion and Future Perspectives

The principal strengths, current limitations, and future directions for developing myxobacteria as reliable biocontrol platforms are summarized in Table 3. Myxobacteria occupy a distinctive position at the intersection of microbial ecology, natural product discovery, and applied biotechnology [24, 25]. They are valuable as model systems for bacterial social behavior and predation [24], as biological resources for biocontrol [34], and as sources of high-value compounds [54]. Their comparison with actinomycetes is justified because both groups are important microbial sources of structurally distinctive natural products [26]. Myxobacteria have yielded compounds with unusual mechanisms and substantial biomedical relevance, including epothilones and tubulysins [56–60]. Their practical exploitation has progressed more slowly, however, because many strains exhibit slow growth, complex developmental behavior, demanding cultivation requirements, and limited genetic tractability [39, 113, 114]. Despite these constraints, their diverse predatory mechanisms [28] enable antagonism against phylogenetically distinct targets, including plant-pathogenic bacteria and fungi, oomycetes, and selected microbial eukaryotes [32, 33, 81–83].

The translational potential of myxobacteria is no longer based solely on laboratory antagonism. Disease suppression has been demonstrated in selected crop pathosystems, with greenhouse and field studies providing evidence of progress toward agricultural deployment [96, 97]. The optimization of *M. fulvus* WCH05 further demonstrates that biocontrol performance can be improved through production and process engineering as well as strain selection [97]. From an applied perspective, myxobacteria offer distinct opportunities as live-cell biocontrol agents and as sources of cell-free bioactive products, in addition to their value for natural-product discovery and biosynthetic engineering [35, 54, 82, 97, 113, 114]. Current agricultural evidence is strongest in selected bacterial and fungal plant-disease systems, although broader field validation across additional pathosystems remains necessary [34, 96, 97]. Potential environmental applications include rhizosphere-level pathogen suppression through microbiome-associated nutrient interference [82] and the emerging control of cyanobacterial targets in aquatic systems [104]. Interactions with soil protists may also have broader implications for microbial food-web and rhizosphere management, although this possibility remains preliminary [33, 100, 101]. Separately, the chemical diversity of myxobacteria continues to support antibacterial, antifungal, antiparasitic, antiviral, and anticancer discovery [25, 54]. Recent predation-oriented metabolomics further illustrates this convergence by integrating ecological strain prioritization with NMR-based natural-product discovery and identifying cystobactamids as effectors that enhance myxobacterial predatory capability across diverse prey [115].

Several barriers must still be addressed before these capabilities can be translated into consistent applications. Improved cultivation and fermentation are needed to increase biomass production and biocontrol performance [97], while preservation, formulation, and scale-up remain important for practical deployment [85]. Genome mining, metabolomics, and synthetic biology should continue to expand access to silent or poorly expressed biosynthetic pathways and improve the tractability of production strains [38, 113, 114]. At the same time, predatory specificity and ecological performance must be evaluated under realistic environmental conditions, with particular attention to persistence, efficacy durability, prey responses, and ecological safety [79, 86, 112]. Progress will therefore require integration of natural-product chemistry, microbial ecology, formulation science, functional genomics, and translational biotechnology [25, 54, 85, 113, 114]. Given the growing demand for environmentally compatible bio-based solutions [2, 4], myxobacteria remain underexploited resources whose mechanistic diversity, chemical richness, and ecological versatility justify continued development as both biocontrol platforms and sources of bioactive compounds [25–27, 39, 54].

## Figures and Tables

**Fig. 1:**
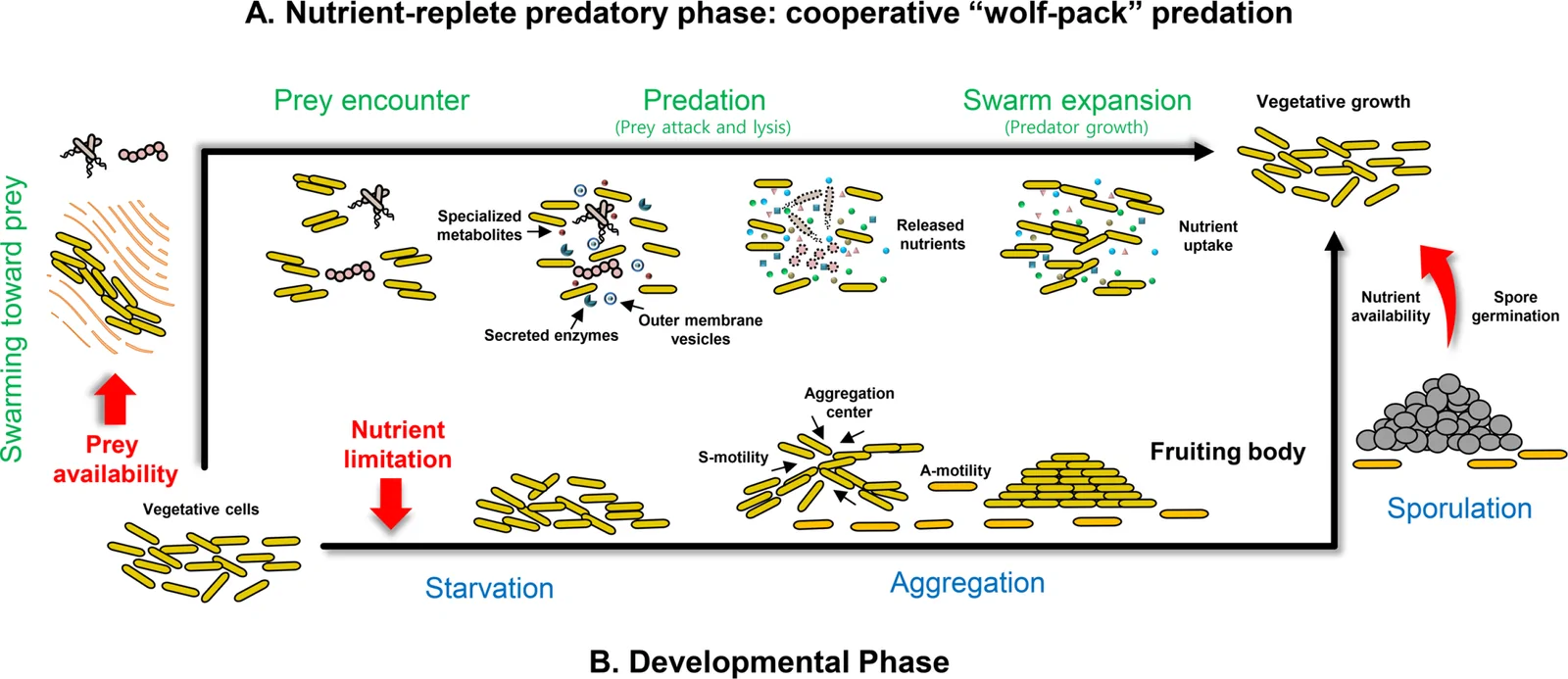
Life cycle of *Myxococcus xanthus* highlighting cooperative “wolf-pack” predation. (**A**) During the nutrient-replete predatory phase, vegetative *M. xanthus* cells move collectively across surfaces, encounter prey, and accumulate at prey-rich interfaces. Prey attack and lysis release prey-derived nutrients that support predator growth and swarm expansion. (**B**) When nutrients become limiting, starvation induces aggregation and fruiting-body development. During development, stress-resistant myxospores form within fruiting bodies, while a subpopulation remains as peripheral rods. When favorable nutrient conditions return, myxospores germinate into vegetative cells and the cycle resumes.

**Fig. 2:**
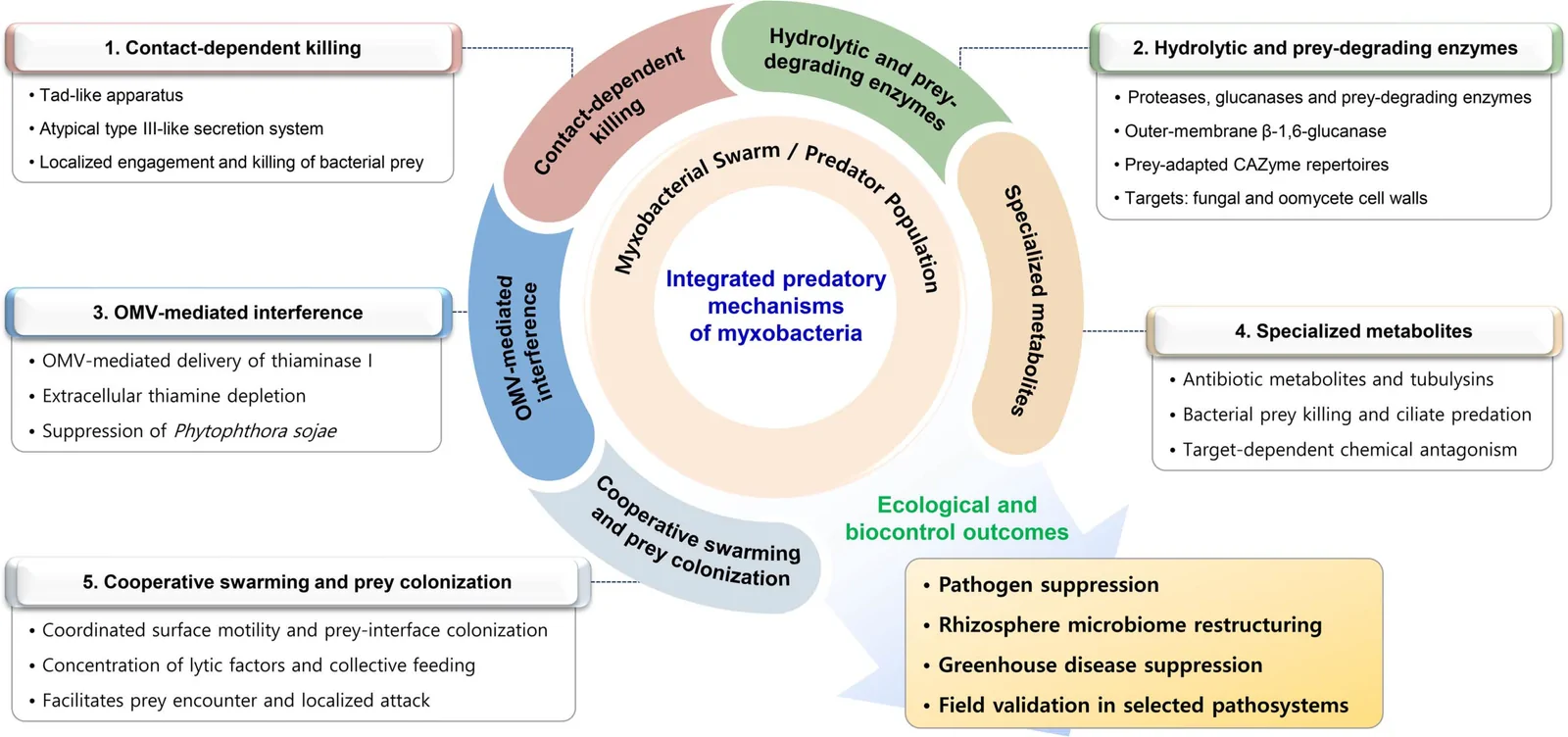
Integrated predatory mechanisms and their ecological and biocontrol outcomes in myxobacteria. The diagram summarizes five major components of myxobacterial predation: contact-dependent killing, hydrolytic and prey-degrading enzymes, outer membrane vesicle (OMV)-mediated interference, specialized metabolites, and cooperative swarming and prey colonization. These mechanisms are presented as the mechanistic basis of myxobacterial predation and are distinguished from the downstream ecological and biocontrol outcomes. The resulting outcomes include pathogen suppression, rhizosphere microbiome restructuring, greenhouse disease suppression, and field validation in selected pathosystems.

**Table 1:** **Representative myxobacterial secondary metabolites and their biological relevance.** Representative secondary metabolites discussed in the subsection “Secondary metabolites and bioactive potential of myxobacteria” are summarized in the order of their appearance in the main text. For each metabolite, the producing myxobacterial taxon, compound class, major bioactivity or molecular target, ecological or translational relevance, and current research significance are indicated. Research significance is described conservatively on the basis of the cited literature and does not represent a formal regulatory or development classification. An ecological role in predation is specified only when directly demonstrated; otherwise, the table indicates pharmacological, mechanistic, or translational relevance without implying a confirmed predatory function.

Metabolite	Producing myxobacterium	Compound class	Major bioactivity / target	Ecological or translational relevance	Current research significance	References
Epothilones	*Sorangium cellulosum*	Macrolides	Microtubule stabilization; anticancer activity	Landmark myxobacterial anticancer scaffold; inspired the development of epothilone analogs, including BMS-247550	Clinically relevant scaffold and derivative development	[56–58]
Tubulysins	*Archangium gephyra* and *Angiococcus disciformis*	Tetrapeptides	Tubulin polymerization inhibition; potent cytotoxicity	Important anticancer scaffold with a directly demonstrated role in ciliate predation	Medicinal chemistry and preclinical research	[33, 59, 60]
Disorazoles	*Sorangium cellulosum*	Macrodiolides	Tubulin-directed microtubule disruption; antimitotic activity	Potent cytoskeleton-targeting anticancer scaffold	Natural-product and mechanistic research	[61]
Chivosazoles	*Sorangium cellulosum*	Macrocyclic polyketides	Actin-cytoskeleton disruption	Representative actin-targeting natural products and chemical-biology probes	Biosynthetic and mechanistic research	[62]
Sorangicin A	*Sorangium cellulosum*	Macrolide-polyether antibiotic	Bacterial RNA polymerase inhibition	Mechanistically distinctive transcription-targeting antibacterial scaffold	Mechanistic antibiotic research	[63]
Myxalamides	*Myxococcus xanthus*	Antibiotic metabolites	Respiratory-chain complex I inhibition	Extend the functional diversity of myxobacterial metabolites to respiratory-chain inhibition	Natural-product and mechanistic research	[64]
Myxopyronin	*Myxococcus fulvus*	α-Pyrone antibiotic	Bacterial RNA polymerase inhibition via the switch region	Antibacterial scaffold with a binding site distinct from that of rifamycins	Antibiotic lead and mechanistic research	[65]
Corallopyronin A	*Corallococcus coralloides*	α-Pyrone antibiotic	Bacterial RNA polymerase inhibition via the switch region	Directly demonstrated contribution to *E. coli* predation; anti-infective lead with anti-*Wolbachia* and antifilarial potential	Advanced preclinical research	[65–67, 72]
Cystobactamids	*Cystobacter* sp. Cb v34	Oligomeric p-aminobenzoate antibiotics	DNA gyrase and topoisomerase inhibition	Predation-enhancing effectors; antibacterial lead potential	Predation mechanism and antibacterial lead research	[68, 115]
Haliangicin	*Haliangium* sp.	β-Methoxyacrylate-type polyketide	Antifungal activity	Structurally distinctive marine-derived antifungal scaffold	Natural-product and biological-activity research	[69]
Aetheramides A/B	*Aetherobacter* sp.	Cyclic depsipeptides	HIV-1 inhibition and cytostatic activity	Illustrate the bioactive chemical diversity of underexplored myxobacterial taxa	Discovery-stage natural-product research	[70, 71]
Antibiotic TA (myxovirescin A)	*Myxococcus xanthus*	Antibiotic secondary metabolite	Prey-selective antibacterial activity; contribution to *E. coli* killing	Directly demonstrated predatory weapon with prey-dependent activity	Mechanistic predation research	[72]

**Table 2:** **Major target groups and current evidence for the biocontrol potential of myxobacteria.** The major target groups discussed in the section “From Predation to Biocontrol Applications” are summarized in the order of their appearance in the main text. Included are representative target organisms, principal predatory or antagonistic mechanisms or active factors, experimental context, current evidence level, and main outcomes. Evidence levels reflect a qualitative assessment of mechanistic resolution, experimental context, breadth of support across strains, targets, or pathosystems, and validation under rhizosphere, pot, greenhouse, or field conditions. These categories are comparative and do not represent a formal quantitative grading system. Importantly, the assigned evidence level refers to the specific pathosystems or experimental models represented by the cited studies and should not be interpreted as applying uniformly to an entire target group.

Target group	Representative target organism(s)	Predatory or antagonistic mechanism / active factor	Experimental context	Evidence level	Main outcome	References
Plant-pathogenic fungi	*Fusarium* spp., *Verticillium dahliae*, *Colletotrichum acutatum*, and *Botrytis cinerea*	Predation-associated antagonism, extracellular enzymes, OMV-mediated delivery of myxothiazole, culture-filtrate-associated antifungal activity, and rhizosphere microbiome reshaping	*In vitro*, rhizosphere/plant, greenhouse, and selected field studies	Field-supported in selected fungal pathosystems	Disease suppression demonstrated in cucumber, cotton, wheat, hot-pepper, and stored-fruit systems, with field validation in selected pathosystems	[34, 83, 89–93, 95, 96]
Plant-pathogenic bacteria	*Ralstonia solanacearum*, Erwinia amylovora	Secreted extracellular proteins and predation-associated antagonism	*In vitro*, pot/greenhouse, and selected field studies	Field-supported in selected bacterial pathosystems	Suppression of tomato bacterial wilt and pear fire blight, with field validation in selected systems	[34, 35, 94, 97]
Oomycetes	*Phytophthora sojae*, *Pythium ultimum*, and *Phytophthora capsici*	Specialized CAZyme system; OMV-mediated delivery of thiaminase I and extracellular thiamine depletion; culture-filtrate-associated antagonism	Mechanistic laboratory and rhizosphere studies; *in vitro* antagonism and greenhouse studies	Mechanistically well supported; field validation remains limited	Prey-specific oomycete predation and rhizosphere-level pathogen restraint demonstrated; greenhouse disease suppression reported, but broader field efficacy remains unvalidated	[32, 82, 89, 99]
Ciliates	*Tetrahymena thermophila*	Tubulysin-dependent prey killing	Mechanistic laboratory study	Direct mechanistic predation evidence; no applied biocontrol validation	Direct demonstration of ciliate prey killing without validated biocontrol application	[33]
Cyanobacteria	*Phormidium luridum* and bloom-forming cyanobacteria	Predation and lysis in aqueous systems	Classical laboratory studies and review-level synthesis	Emerging; laboratory evidence without field-scale validation	Direct antagonism of selected cyanobacterial prey demonstrated experimentally; control of natural blooms remains unvalidated	[104–106]
Nematodes	*Caenorhabditis elegans* (model nematode)	*In vitro* nematicidal activity of myxobacterial extracts; DKxanthene-dependent suppression of *C. elegans*	*In vitro* extract-based and co-culture assays using *C. elegans*	Preliminary; *in vitro* model-nematode evidence only	Antinematodal activity demonstrated against *C. elegans*; direct predation and control of plant-parasitic nematodes remain unvalidated	[110, 111]

**Table 3:** **Current strengths, limitations, and future directions for developing myxobacteria as biocontrol platforms.** This table summarizes the major strengths, current limitations, and future research directions discussed in the subsection “Opportunities and bottlenecks for field application” and the section “Conclusions and future perspectives”, with emphasis on key translational considerations including mechanistic diversity, disease suppression potential, formulation and deployment challenges, and biosafety or regulatory issues. The categories are conceptual and interconnected, and the cited references are representative rather than exhaustive.

Aspect	Current strength	Current limitation	Future direction	Key references
Target breadth	Evidence extends across bacterial prey and selected fungal, oomycete, ciliate, and other eukaryotic targets	Breadth does not automatically guarantee field consistency or target selectivity	Define target range more rigorously across ecologically realistic systems	[27, 28, 32, 33, 81, 83]
Mechanistic diversity	Predatory mechanisms include cooperative swarming, contact-dependent killing, hydrolytic enzymes, OMV-mediated interference, specialized metabolites, and nutrient competition	Relative contribution of each mechanism remains insufficiently resolved *in situ*	Integrative spatial and ecological mechanism studies	[27–32, 72, 76, 82–84]
Disease suppression potential	Strongest field-oriented support in selected bacterial and fungal plant-disease systems; strong mechanistic and rhizosphere-level support for oomycetes	Large-scale and multi-location validation remains limited	Expand greenhouse-to-field pipelines and standardized validation frameworks	[32, 35, 82, 92, 93, 96, 97]
Natural product value	Rich source of bioactive natural products with antibacterial, antifungal, and anticancer relevance	Many compounds remain underexplored functionally and biosynthetically	Genome mining, metabolomics, and biosynthetic engineering	[23, 25, 37, 38, 54, 113–115]
Formulation and deployment	Greenhouse and field validation in selected pathosystems, with emerging development of multiple application formats and fermentation optimization	Live-cell and cell-free approaches have distinct requirements for production, formulation, stability, delivery, persistence, and activity retention	Develop application-specific production, formulation, and delivery strategies and expand multi-location field validation	[85, 86, 96, 97, 112]
Biosafety and regulatory translation	Potential to complement or reduce reliance on conventional chemical pesticides	Non-target effects, environmental persistence or fate, and regulatory requirements remain insufficiently defined	Application-specific ecological risk assessment and regulatory framework development for live-cell and cell-free products	[85, 86, 112]
